# Heart Failure in a Dedicated Outpatient Clinic: Results after 58 Month Follow-Up. Can it be Enough?

**Published:** 2014-12-19

**Authors:** M Mirra, G Vitulano, N Virtuoso, N Tufano, F D’Auria, S De Angelis, R Giudice, A Lambiase, A Gigantino, F Piscione

**Affiliations:** 1 Department of Medicine and Surgery, University of Salerno, Salerno, Italy.; 2 Department of Emergency, University Hospital of Salerno, Salerno, Italy.; 3 Department of Medical and Surgical Cardiology, University Hospital of Salerno, Salerno, Italy. (fpiscione@unisa.it)

**Keywords:** *heart failure*, *hospital outpatient clinic*, *clinical practice guideline*

## Abstract

Incidence of chronic heart failure (HF) is rapidly increasing, approaching a 10 per 1000 rate after 65 years of age. In the last decades, despite pharmacological, interventional and supportive innovations, HF prognosis remained poor, with about 30% of death within one year from the diagnosis. Current guidelines recommend for these patients management programs providing follow-up through dedicated outpatient clinic. Limits of these programs are represented by great difficulties in getting patients adherence, being still too elevated the rate of abandonments. In this paper, we analyzed the impact of 58 months of activity in our dedicated to heart failure outpatient clinic on mortality, hospitalization and abandonment rate. 477 HF patients (346 M, 72.5%, mean age 69.6 years) were enrolled. Mean follow-up and visit were 18.2 and 2.6 months respectively. Total mortality rate was 11.5%, 4% of patients per year. Total hospitalizations for acute HF were 212 and, among all patients left in follow-up, the number of hospitalizations for acute de-compensation significantly decreased from 0.49/patient/year before enrollment to 0.29/patient/year during follow-up (p=0.015). Patients who abandoned outpatient clinic were 94 (19%, 1 abandonment every 23 days), mostly observed over the first months of activity. In conclusion, our patients experienced a major decrease in rates of acute de-compensation and need of in-hospital admissions.

## I. INTRODUCTION

Incidence of chronic heart failure (HF) still to be increased, approaching 10 per 1000 population after 65 years of age; in white man, the annual rates of new HF events are 15.2 every 1000 individuals between 65 to 85 years, and 65.2 for those ≥85 years of age [1]. In the last decades, despite pharmacological, interventional and supportive innovations, HF prognosis remained poor, with about 30% of death in one year from the diagnosis [2]. Heart failure management summarize more than 2% of total healthcare costs, the most of which are related to hospitalizations [3]. According to current ESC guidelines [4] HF patients should be involved in management programs lead by a multidisciplinary team in an outpatient clinic, providing for each patient an accurate diagnosis, the appropriate evidence-based therapy and education for both patients and their carers. This “holistic” approach leads to a significant reduction in hospitalization rate, thus improving both survival and quality of life [5–6]. Nevertheless these programs are frequently insufficient, leading to inadequate support and suboptimal treatment. Particularly adherence of patients at follow-up is often poor, with still too elevated percentage of abandonment. Less is known about causes of these poor outcomes, and which aspects need more sensitive improvements [7–10]. Aim of this paper is to describe our 58 months experience in a dedicated HF outpatient clinic and its impact in terms of hospitalizations, abandonments and deaths. A service led by a cardiologist and a specialized nurse, in which patients can easily get a visit or telephone contact.

## II. METHODS

### Study population

The clinical records of 477 patients admitted to our dedicated to heart failure outpatient clinic in the University Hospital of Salerno, from January 2009 to October 2013, were retrospectively reviewed. Most of these patients have been enrolled at time of discharge from hospitalization in our institution for HF. All data regarding medical history and anthropometric features, variation of functional class, in-hospital admissions and causes, deaths and abandonments of the follow-up were reported. For any patient medical controls were planned at 1 month and at least every 6 months, further patients access were performed according to clinical judgment. In every visit the anthropometric parameters were evaluated and a full cardiological evaluation, EKG, pressure measurement, and a critical revision of medical therapy were performed; furthermore a personalized counseling in order to improve pharmacological adherence was performed. A transthoracic echocardiography was performed in order to evaluate global cardiac function. Any other diagnostic exam, as well as specialist medical consultancy, were performed according to clinical judgment.

### Statistical analysis

Continuous variables have been expressed as the mean ± standard deviation and categorical variables as percentage. The analysis of difference between the two groups has been performed by student’s unpaired t-test for continuous variables and χ^2^ test for categorical variables. Wilcoxon signed-rank test was employed to compare number of hospitalization/patient/year related to heart failure between a period of a year before enrollment and during follow-up. The same test was used to compare functional class (NYHA) at time of enrollment and during follow-up. All values have been analyzed bilaterally and p-values <0.05 have been considered significant. All statistical analysis have been performed using SPSS for Windows, version 19.0 (SPSS Inc., Chicago, IL, USA).

## III. RESULTS

**Figure 1.**
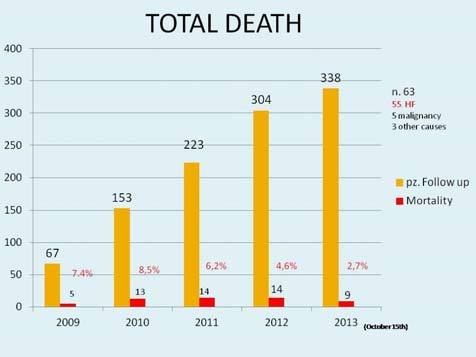
**Percentage mortality reduction from 2009 to 2013, with a progressive increase of followed patients. P for trend = n.s.**

**Figure 2.**
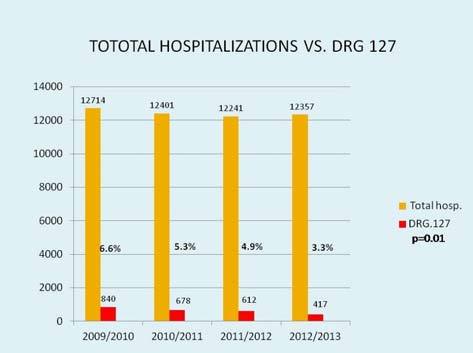
**Comparison between overall hospital admission and admission for diagnosis-related groups (DRG) 127 (heart failure). P for trend = 0.01.**

**Figure 3.**
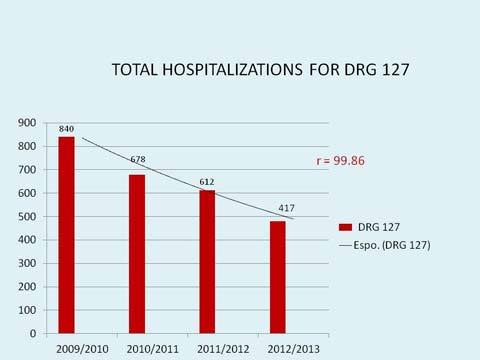
**Linear correlation between total hospital admission and diagnosis-related groups (DRG) 127 (r=99.86) and DRG 127 and hospitalization (r=99.45).**

Four hundred seventy seven HF patients (346 M, 72.5%, mean age 69.6 years) were enrolled in 58 months of clinical activity. The predominant HF etiology is ischaemic, followed by idiopathic forms, hypertensive forms, post valvular disease and post-myocarditis beeing 53.3, 18.2, 13.9, 8.6 and 1.1% respectively. The majority (68%) were NHYA I–II class. Mean follow-up was 18.2 months and mean visit interval was 2.6 months. Patients which abandoned outpatient clinic were 94, 19% of followed patients, with an average abandonment every 23 days. Total patients deaths were 55: a percentage of 11.5%, equivalent to 4% of patients per year, a death every 30 days, on average (Fig. 1). Total re-hospitalization for acute HF were 212. Among all patients left in follow-up the number of hospitalizations for acute de-compensation significantly decreased from 0.49/patient/year before enrollment to 0.29/patient/year during follow-up (p=0.015). Total readmission for heart failure decreased significantly from 840 to 417 (p= 0.01) over the follow-up period (Fig. 2). Mortality rate decreased from 8.5% in the 2009 to the 3.3 % in the 2013 (p = n.s.). Patients followed need an hospitalization every 45 months of follow-up, on average. Most of patients (167 patients, equivalent to 52.2% of total, and 78.8% of patients left) in follow-up did not need in hospital admissions for heart failure during follow-up. Only 45 patients (equivalent to 21.1% of patients left in follow-up and 14.1% of total patients) needed one or more hospitalization for this cause. No significant differences in NYHA class modification were observed (p =0.839), eventhough an interesting trend was pointed out, with a reduction of NYHA class III–IV from 42% in 2009 to 27% in the last year. Another interesting finding is the constant reduction of hospital admission for diagnosis-related groups (DRG) 127 (heart failure). Total hospital admission for HF in 2009 represented 6.6% of all admission, and after 3 years of ambulatory activity was appreciated a reduction till 4.9% of the total amount, with a mean absolute reduction of 228 admission/year. A linear correlation between total hospital admission and DRG 127 (r=99.86) and DRG 127 and hospitalization (r=99.45) were also observed (Fig. 3). One, two and three or more comorbidities, were respectively observed in 203 patients (64%), 57 patients (18%) and 10 patients (3.1%). The most common comorbidity was diabetes, followed by chronic kidney disease and respiratory diseases. Total deaths were 55, 11.5% of our population; among them 45 patients (83%) showed at least one comorbidity, and 11 (20%), 42 (76.3%) and 2 (3.4%) were in II, III and IV NYHA class, respectively.

## IV. DISCUSSION

The actually recommended programs for chronic care management of heart failure provide, among other things, structured follow-up in a dedicated outpatient clinic. Our service ensure a dedicated follow-up, with patient education, regular control of pharmacological treatment, and easy access to care and visit. But management programs, on the other hand, should be multidisciplinary with intervention of different professional figures, and primarily providing a chain-of-care delivered by various services of the healthcare system [11–19]. Unfortunately we have not been able yet to create appropriate links and collaboration with primary care providers and other figures. First of all the link between the heart team and the general practitioner is crucial, thus ensuring the continuity of cares; unfortunately it is not always possible. Nevertheless, some clinical outcomes were very encouraging: only one fifth of patients needed an hospitalization due to heart failure during follow-up, whereas less than 1/7 seven died during this period. A notable aspect is that most patients were enrolled for follow-up at time of discharge, and all have been assessed by cardiologists. Another possibility of enrollment is by using our dedicated internet page, in which the general practitioner or colleagues from community hospitals could require a specialist visit directly from their workplace. Since the availability of internet patient even more patients are sent from peripheral centers, making our outpatient clinic ever more rooted in the territory. Furthermore, percentage of abandonments was also quite limited: about 1/5 abandoned the follow-up. Not equally appreciable was datum related to frequency of visits: each patient came for visit every 96 days, on average. This in spite of our efforts to make easy access and telephonic contact with nurse and cardiologist. Recently we have postulate the possibility of using a new marker for the characterization of heart failure and preliminary results are encouraging [20]. Percentage of patients lost for follow-up and frequency of visit, are two aspects strongly linked to “chain of care” of healthcare. Moreover these aspects could probably affect other clinical outcome (especially mortality and hospitalization). NHYA functional class was not significantly modified during the follow-up, but this aspect was not surprising because patients shows a prevalent low NYHA class at enrollment time; an indirect evaluation of these finding reveals the prolonged maintenance of low NYHA class and a relative good clinical condition. Global NYHA class III–IV reduction from 42% in 2009 to 27% of overall population in 2013 are mainly related to drugs compliance and home monitoring. Finally, analyzing global mortality data more than 80% of death were in advanced NYHA class and we observed a progressive rate reduction from 8.5% in the 2009 to the 4.3% in the 2013. Our mortality rate is lower than IN-CHF registry, in which it was reported as about 10% [21]. It could possibly derive from either the enrollment modality, with an higher prevalence of lower NYHA class (I–II) and from the short term observation.

## V. CONCLUSIONS

The results of 58 months of work in our dedicated outpatient clinic confirm value of management programs for patients with HF, but suggest that still efforts are needed to improving adherence at these programs. Our patients experienced sensitive decrease in frequency of acute de-compensation and need of in-hospital admissions. Perhaps this phenomenon could be mitigated through a more efficient organization of our outpatient clinic and/or involvement of other territorial institutions, with different professional figures, in order to improve satisfaction degree and communication tools with our patients. A more structured and comprehensive strategy is needed for improve loyalty of our patients, and consequently clinical outcomes. The improvement of the internet available services could be an effective strategy to increase patient adherence.
